# Intussusception After Roux-en-Y Gastric Bypass: Correlation Between Radiological and Operative Findings

**DOI:** 10.1007/s11695-022-06377-2

**Published:** 2022-12-07

**Authors:** Hassan Zaigham, Mikael Ekelund, Daisy Lee, Olle Ekberg, Sara Regnér

**Affiliations:** 1 Department of Clinical Sciences, Malmö, Section of Surgery, Lund University, Skåne University Hospital, Malmö, Sweden; 2 Department of Translational Medicine, Diagnostic Radiology, Lund University, Skåne University Hospital, Malmö, Sweden

**Keywords:** Abdominal pain, Bariatric surgery, Computed tomography, Gastric bypass, Intestinal obstruction, Intussusception

## Abstract

**Introduction:**

Intussusceptions diagnosed on computed tomography (CT) scans in Roux-en-Y gastric bypass (RYGB) patients could cause serious small bowel obstruction (SBO) or be an incidental finding. The objective of this study was to correlate radiological findings with clinical outcomes to differentiate intussusceptions requiring emergent surgery for SBO.

**Methods:**

A search for acute abdominal CT scans reporting intussusceptions in RYGB patients between 2012 and 2019 at Skåne University Hospital, Malmö, Sweden, retrieved 35 scans. These were independently reevaluated by two radiologists for the length and location of the intussusception, whether oral contrast passed through, proximal bowel dilatation, and signs of internal herniation. Clinical outcome in terms of emergency surgery and the diagnosis was determined through chart review.

**Results:**

Out of 35 acute patients, 9 patients required emergency surgery within 24 h. Intussusception caused SBO in five patients, and one patient had an internal herniation, while three patients had unremarkable findings. Eight patients were evaluated for intermittent pain with five unremarkable laparoscopies, while 18 patients had intussusceptions as incidental findings. Intussusception length on CT as measured by radiologists O.E. and D.L. predicted acute bowel obstruction (*p* = .014 and *p* < .001). A 100 mm threshold predicted bowel obstruction with a sensitivity of 80% and 100% and a specificity of 93% and 86% by radiologists O.E. and D.L., respectively. Proximal bowel dilatation predicted SBOs of any cause as well as SBO caused by an intussusception (all *p* < .05).

**Conclusion:**

Intussusception length > 100 mm on CT in RYGB patients is an easy and valuable sign indicating SBO that may require emergent surgery.

**Graphical Abstract:**

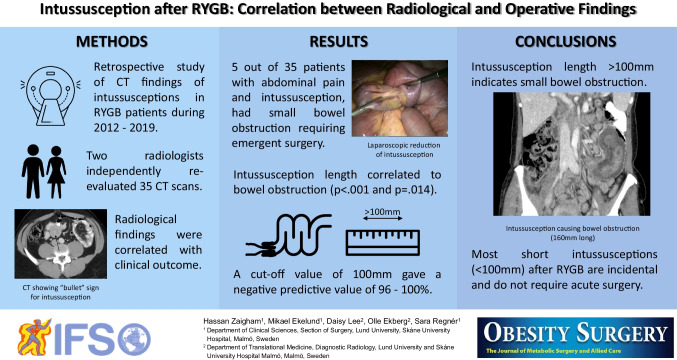

**Supplementary Information:**

The online version contains supplementary material available at 10.1007/s11695-022-06377-2.

## Background

An abdominal computed tomography (CT) scan is the imaging study of choice in evaluating acute abdominal pain in a Roux-en-Y gastric bypass (RYGB)-operated patient. However, due to the high frequency of unexplained chronic abdominal pain in this population, the imaging findings may be challenging to correlate clinically, and further investigation may require a diagnostic laparoscopy [1, 2]. One typical example is a CT scan reporting a jejunal intussusception, as the clinical implication is uncertain with varying degrees of symptoms. Intussusception is a rare cause of small bowel obstruction (SBO) in the adult population and is typically caused by a lead point in the form of a polyp, tumor, or adhesion [3]. In RYGB patients, where the incidence is much higher, a lead point is seldom found, and clinical experience has shown that jejunal intussusceptions may often occur and resolve spontaneously. We have not found any studies that have reported the outcome of CT diagnosed intussusceptions in RYGB patients. In 2018, Tan et al. evaluated the clinical outcome of CT diagnosed intussusception in a general adult population in an attempt to construct an ambitious clinical scoring system [4]. However, neither of the two major factors found in their study, a lead point and colonic involvement, apply to the RYGB population where most intussusceptions are jejunal without a lead point.

Clinical experience has shown that intussusceptions in RYGB patients often reduce spontaneously, but at other times, it may cause intestinal obstruction with ischemia if left untreated and therefore requires emergency surgery. The clinical challenge is to differentiate those in need of emergent surgery. Presently surgeons rely on the clinical presentation in correlation to the radiological finding, when determining whether an intussusception requires emergent surgical exploration or not.

The mechanism causing an intussusception in RYGB patients is poorly understood, with theories of the jejuno-jejunal (j-j) anastomosis acting as a lead point or that the peristaltic movement becomes asynchronous because of the Roux limb and therefore causes the bowel to invaginate into itself [5]. Intussusceptions in RYGB patients are seen in different parts of the small bowel; however, they often appear in proximity to the j-j anastomosis. The surgical treatment is debated, and there is no consensus about whether a reduction is sufficient or an enteropexy, resection, or reconstruction of the j-j anastomosis should be performed [6]. Advocates for the asynchronous peristaltic wave theory argue that the anti-peristaltic side-to-side j-j anastomosis is to blame and therefore suggest that the anastomosis is reconstructed either as an end-to-side or isoperistaltic side-to-side anastomosis. There are multiple (> 30) individual case reports in the literature but only a few case series [7–11].

RYGB in Sweden is performed laparoscopically with an antecolic and antegastric approach [12]. The prevalence of RYGB-operated patients is high in the study region, as about 80/100,000 inhabitants were operated annually during the last decade [13, 14]. In a retrospective review, we reported that two out of 300 consecutive RYGB patients admitted for acute abdominal pain to our University Hospital required surgery for SBO caused by an intussusception [15]. However, the incidence of radiologic findings of intussusception on acute abdominal CT scans is much higher.

To the best of our knowledge, there are no earlier studies reporting a correlation between radiological findings of intussusceptions and clinical outcomes in RYGB patients. The aim of this study was to identify imaging findings of intussusceptions on abdominal CT in RYGB patients and correlate them to clinical outcomes, in particular the need for emergency surgery for intussusception with SBO.

## Method

### Database Search

A retrospective search for acute abdominal CT scans performed at Skåne University Hospital in Malmö, Sweden, between 2012 and 2019 was used for the identification of patients. All CT scans were performed on one of several helical CT machines. Helical slices of 1 mm thickness were obtained and reconstructed in transverse, coronal, and sagittal planes. Contrast enhancement using Omnipaque (GE Healthcare AB, Danderyd, Sweden) was administered intravenously and/or orally as appropriate. An electronic search in the radiology database RIS/PACS (Sectra AB, Linköping, Sweden), using the terms “GBP” or “gastric bypass” in the referral and “invag” (short for invagination, i.e., intussusception in Swedish) in the report, was performed. The search results were reviewed manually, and reports of negative findings, i.e. where an intussusception was negated, were excluded as well as patients that had previously had their Roux-en-Y anatomy reversed.

### Radiological Reevaluation

Reevaluation was performed using a predetermined protocol by two independent radiologists unaware of the clinical outcome, with 42 and 1 year(s) experience since board approval, respectively. The results and inter-rated reliability were calculated for each radiologist separately. The use of intravenous and/or oral contrast was noted, and the diagnosis of intussusception was verified. The protocol included five variables:

1. Is the intussusception located in the left upper quadrant (LUQ)?
2. Does the intussusception cause proximal bowel dilatation?
3. Has positive oral contrast passed through the intussusception?
4. Measurement of the length of the intussusception in millimeters (mm).
5. Signs of an internal herniation?

As the j-j anastomosis, usually located in the LUQ, is commonly involved in the intussusception, variable 1. was investigated as a proxy for an intussusception near the j-j anastomosis. Variables 2. and 3. were investigated as common signs of SBO.

### Medical Chart Review

Medical records and operation charts were retrospectively reviewed by a consultant surgeon, H.Z. Admission symptoms of acute and chronic abdominal pain, nausea, and vomiting, time since RYGB surgery, length of stay, surgery performed, and diagnosis at discharge were extracted. Weight in kilograms (kg) and height in meters (m) at admission were obtained and body mass index (BMI) in kg/m^2^ was calculated. Any hospital visits and readmissions within 1 year were investigated for recurrence of intussusception. Electronic records were accessible from all surgical wards in the Region of Skåne serving the population.

### Categorization of Intussusceptions

Patients were grouped into the following three categories in terms of requiring acute surgery and discharge diagnosis:

1. Acute intussusception. Defined as patients with a clinical presentation requiring emergent surgery performed within 24 h of admission.
2. Intermittent intussusception. Defined as a surgical admission with a discharge diagnosis of intussusception but not requiring emergent surgery within the first 24 h of admission.
3. Incidental intussusception. Defined as patient that either did not require admission or was discharged with a different diagnosis when the intussusception was considered an incidental finding.

### Statistical Analysis

All data were stored and analyzed using SPSS version 28 (IBM Corporation, Armonk, NY, USA). Results are expressed as a median with minimum and maximum values. Kruskal–Wallis non-parametric test was used for group comparisons of continuous values, while Fisher’s exact test was used for comparison of categorical variables. All group comparisons were unpaired. A *p* value of ≤ .05 was considered statistically significant. Spearman’s rho (ρ) non-parametric test was used for the strength of association between two non-normally distributed scale variables, and Cohen’s kappa (κ) was used for inter-rater reliability of qualitative variables.

## Results

**Fig. 1:**
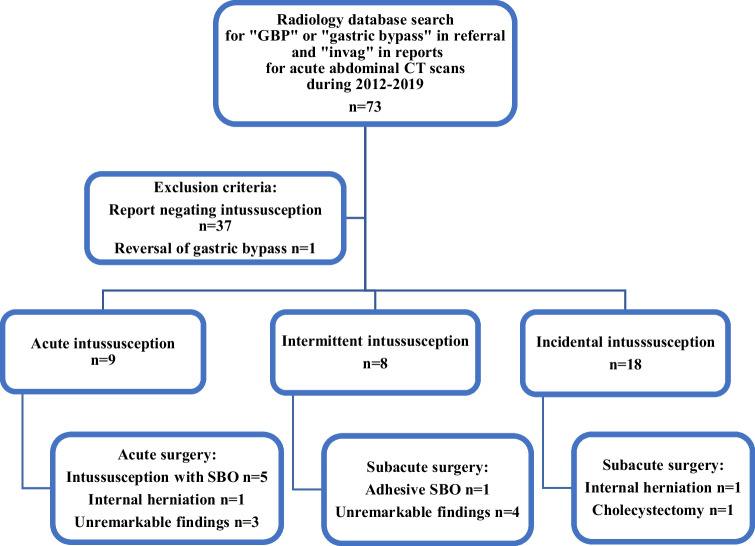
Flow chart of inclusion and outcome of the patient cohort. CT, computed tomography; GBP, gastric bypass; SBO, small bowel obstruction

The radiological database search yielded 73 CT scans. After exclusion of 37 scans negating intussusception findings and one patient that had a gastric bypass reversal, 35 CT scans were included in the study, and patients were divided into the aforementioned categories as shown in the flowchart (Fig. 1).

### Acute Intussusception

**Fig. 2:**
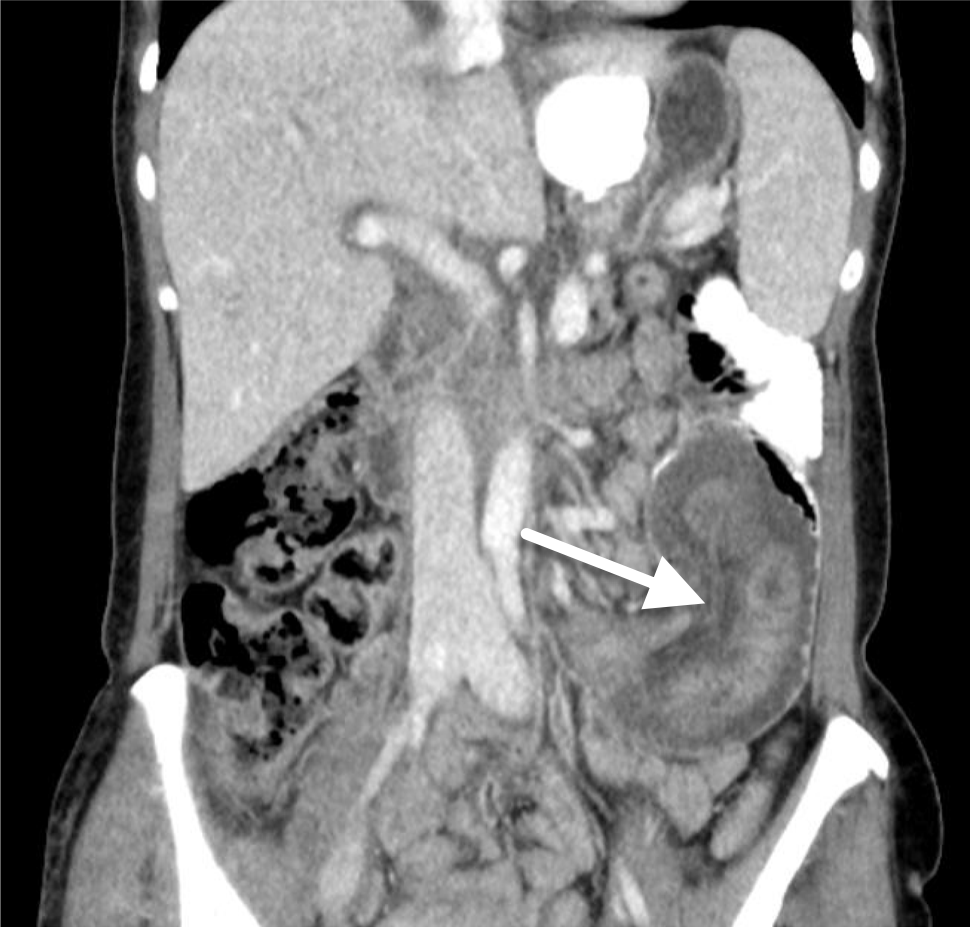
Computed tomography scan illustrating a 160-mm-long intussusception

Nine patients had severe acute symptoms at presentation and required emergent surgery. One patient presented with peritonitis, while the others had opiate-resistant pain. Acute surgery revealed persistent intussusceptions requiring an active reduction in five patients. Three of these patients had ischemic and obstructed bowel caused by strangulated intussusception near the j-j anastomosis, two of which had ischemic perforations. They were treated with bowel resection and remodeling of the anastomosis. The two other patients had viable intussusceptions causing obstruction treated with reduction without bowel resection. One of these patient’s CT scans showed a 160 mm long intussusception (Fig. 2). The successful laparoscopic reduction is demonstrated in the Supplemental Video.

### Intermittent Intussusception

Eight patients were observed in-hospital and investigated for intussusception as the cause of their abdominal pain. Five patients underwent subacute diagnostic laparoscopies. Four patients had largely unremarkable laparoscopies; however, two patients underwent remodeling of their j-j anastomosis to an isoperistaltic side-to-side anastomosis as a preventive measure to reduce risk of recurrence. The fifth patient required open surgery with adhesiolysis for adhesive SBO occurring a year after an elective j-j anastomosis reconstruction. Three patients had intermittent symptoms that subsided and were discharged with outpatient follow-up. One of these had 4 months prior to the admission had an elective remodeling of her j-j anastomosis.

### Incidental Intussusception

In a majority of patients (18/35), the intussusceptions were treated as incidental findings. Six patients did not require in-hospital care. The remaining twelve patients were treated for various surgical diagnoses ranging from appendicitis to internal herniation, while seven patients received a diagnosis of unspecified abdominal pain. Two patients underwent subacute surgery, an internal herniation, and a subacute cholecystectomy following biliary pancreatitis.

The study cohorts’ demographics with presentation symptoms and admission data for each category as well as for intussusceptions causing SBO compared to the rest are presented in Table 1. The five patients with an intussusception causing SBO did not differ significantly from the rest of the cohort in terms of age (*p* = .069), BMI (*p* = .071), or time since RYGB surgery (*p* = .873). Neither were any differences observed in terms of presenting symptoms.

**Table 1:** Study cohorts’ demographics, presentation symptoms, and admission data presented categorized by the clinical category or for bowel obstruction

	Intussusception				
	Acute	Intermittent	Incidental	Causing bowel obstruction	Not causing bowel obstruction
Total	9	8	18	5	30
Sex					
Female Male	9 0	7 1	15 3	5 0	26 4
Age (years)	41.3 (23–57)	34.7 (20–47)	38.2 (28–52)	41.3 (36–55)	35.9 (20–57)
BMI (kg/m^2^) *Missing data*	24.0 (19–27) 0	27.2 (22–41) 0	26.5 (21–33) 4	23.0 (19–27) 0	25.6 (21–41) 6
Time since RYGB (months) *Missing data*	32.4 (5–81) 0	25.1 (10–79) 0	30.0 (5–107) 0	32.4 (12–81) 0	26.6 (5–107) 0
Symptoms					
Acute abdominal pain Chronic pain Vomiting Nausea	9 4 4 7	8 4 2 5	18 5 5 12	5 2 3 4	30 11 8 12
Hospital stay (days)	7 (2–17)	6.5 (3–63)	2.5 (2–6)^*^	9 (3–17)	3.5 (2–6)^*^
(Re)admission					
Within 30 days Within 1 year	2 4	1 5	2 2	1 2	4 9
Intussusception recurrence					
Within 30 days Within 1 year	0 1	0 1	0 0	0 0	0 2

Data are presented as count (*n*) or median (minimum–maximum). ^*^ Excluding the six patients that were not admitted

In summary, 40% (14/35) of patients underwent surgery on the suspicion of intussusception when only five patients had an intussusception causing acute SBO. Seven surgeries showed unremarkable findings; however, four patients had procedures to prevent a recurrence and remained free from recurrence at a 1 year follow-up. Two patients with unremarkable laparoscopies without preventive intervention had recurrences of intussusceptions within a year and then had surgical remodeling of their j-j anastomoses. Furthermore, both patients that required surgery for an internal herniation had radiological recurrence of an intussusception 15 and 16 months later, respectively. Patients with incidental intussusception did not have any intussusception recurrence within a year.

### Radiological Reevaluation

All 35 CT scans were considered sufficient in quality and used intravenous contrast enhancement, while additional positive oral contrast was administered for 32 acquisitions. The radiologists did not verify an intussusception in one scan each. One radiologist instead diagnosed a suspected internal herniation. Neither of these two patients required surgery or had a recurrent intussusception within a year.

The radiological reexamination of the remaining 34 CT scans are presented in Table 2 where patients with intussusception causing are compared to self-limited intussusceptions. Most intussusceptions were located in the LUQ without any correlation to SBO. Positive oral contrast was seldom used in the most acute setting. However, proximal bowel dilatation significantly correlated to intussusception with SBO for both radiologists O.E. and D.L. (*p* = .001 and *p* = .044). It also correlated to SBO of any cause, when including patients with internal herniation and adhesive SBO (*p* = .041 and *p* = .035).

**Table 2:** Results of the radiological reexamination of acute abdominal computed tomography scans reporting an intussusception

	Radiologist O.E			Radiologist D.L		
	Intussusception			Intussusception		
	Causing bowel obstruction	Not causing bowel obstruction	*p* value	Causing bowel obstruction	Not causing bowel obstruction	*p* value
Computed tomography scans	5	29		5	29	
Length of intussusception (mm)	113 (30–160)	42 (18–110)	.014	160 (115–220)	30 (14–240)	< .001
Proximal bowel dilatation?	5	5	< .001	5	12	.044
Intussusception in the left upper quadrant?	3	20	1.0	3	25	.205
Passage of oral contrast?	1^*^	29	.065	0^*^	22	.077
Signs of internal herniation?	0	0	n/a	0	2	n/a

Data are presented as count (*n*) or median (minimum–maximum). Mann–Whitney *U* non-parametric test was used for statistical comparison of the length of intussusception and Fisher’s exact test was used for qualitative variables. ^*^ Three patients were not administered any oral contrast

**Fig. 3:**
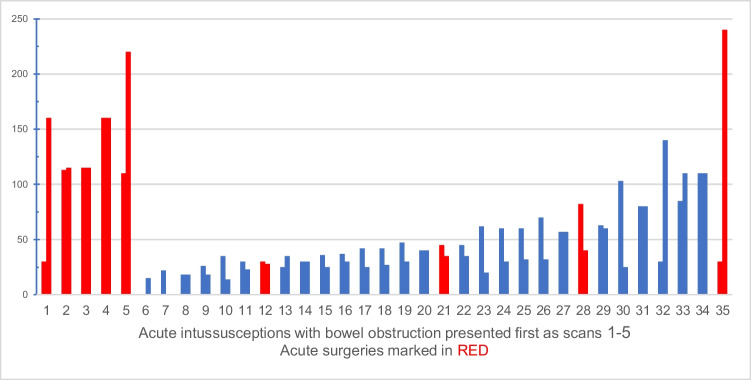
A bar chart of the intussusception length on CT in millimeters by radiologists O.E. and D.L. displayed in incremental size order. Acute surgeries are marked red, and the five patients with bowel obstruction are presented first (1–5). CT, computed tomography

The measured length of the intussusception was significantly longer for patients with intussusception causing SBO for both radiologists O.E. and D.L. (*p* = .014 and *p* < .001) as illustrated in Fig. 3. Using a threshold of 100 mm to differentiate intussusceptions causing SBO gave 80% and 100% sensitivity and 93% and 86% specificity for radiologists O.E. and D.L., respectively, as calculated from the contingency in Table 3. The positive predictive value became 67% and 56%, respectively, and the negative predictive values were 96% and 100%, respectively. A Spearman’s rho of ρ = .429 for intussusception length showed a moderate correlation between the two radiologists’ measurements. Using the 100 mm threshold for intussusception length, an inter-rater reliability comparison was made with a Cohen’s kappa of κ = .574, showing a moderate correlation.

**Table 3:** Association between measured intussusception length on CT and the intussusception causing bowel obstruction

Intussusception length on computed tomography	Bowel obstruction	No bowel obstruction	Total
a. Radiologist O.E.:			
Long (> 100 mm)	4	2	6
Short (≤ 100 mm)	1	27	28
Total	5	29	34
b. Radiologist D.L.:			
Long (> 100 mm)	5	4	9
Short (≤ 100 mm)	0	25	25
Total	5	29	34

## Discussion

The current study provides new knowledge. In particular, in addition to common signs of SBO such as proximal bowel dilatation, intussusception length on CT significantly correlated to SBO requiring emergent surgery. Even the relatively few scans provided data that clearly indicate that long intussusceptions (> 100 mm) have an increased likelihood of causing a SBO requiring emergent surgery. Likewise, shorter intussusceptions have a greater chance of being self-limiting. We have further shown that intussusception length may be determined irrespective of the radiologist’s experience as both had a moderate agreement and showed statistical significance in determining SBO. We therefore propose that a threshold intussusception length of 100 mm be used as a valuable tool with a high sensitivity and specificity for differentiating patients with SBO.

In contrast, neither the location of the intussusception nor whether oral contrast passed the intussusception had any correlation to the clinical outcome. It was noted that oral contrast use was disregarded in most emergent scans, presumably to prevent the time delay in administrating it. Hence, this factor could not be evaluated. But there is no indication that it would be of value.

It is also important to note that patients may experience abdominal pain from intermittent intussusceptions and revisional surgery is often considered. However, determining the optimal treatment for intermittent intussusceptions requires further studies and should preferably be handled with care by bariatric surgeons in an elective or semi-elective setting. In our study, all surgical interventions seemed to reduce the risk of recurrence. It is noted, however, that a majority of patients with intermittent intussusceptions had readmissions for abdominal pain within a year (Table 1). During a 3-year follow-up, half (4/8) had undergone revisional surgery, and one patient had a RYGB reversal. Unfortunately, this did not seem to reduce symptoms, as all but one patient had recurrent visits and/or readmissions for abdominal pain during a 3-year follow-up (data not shown). However, the small cohort does not allow for any conclusions and the best surgical strategy for these patients has not yet been thoroughly studied.

It is further noted that the investigated cohort had a relatively low BMI (median 25.0) and those with SBO even lower (median 23.0). A comparison was therefore made to our previously published cohort of 300 RYGB patients admitted for acute abdominal pain that after excluding two patients with intussusceptions had a significantly higher median BMI of 28.7 (*p* = .002) [15]. We speculate that perhaps a thin and floppy mesentery may contribute to the risk of intussusception. There was however no difference in terms of total weight loss (*p* = .946, data not shown).

Although the clinical decision to perform emergency surgery always relies on a combination of factors, our results show that a longer intussusception is more likely to require surgery, and this can be an important aid in the decision-making. It is feasible and logical to believe that shorter intussusceptions have a greater chance of spontaneously resolving, which our data is first to prove.

Despite being a retrospective single-center study, the relatively large cohort provides new clinically important and encouraging information. The follow-up period of 12 months covered all emergency department visits and surgical admissions within our region and would therefore ensure that symptomatic intussusceptions would not be missed, indicating that two thirds of these patients did not require either elective or acute surgery but had incidental findings that spontaneously resolved. Any future validating studies should preferably be multicenter studies with longer follow-up in order to investigate later recurrences.

## Conclusion

This study introduces a new radiological sign to aid in clinical decision-making in RYGB patients with a CT diagnosis of intussusception. We have shown that an intussusception length of > 100 mm is an easy and useful radiological sign to alert surgeons of patients that may require emergency surgery for SBO. We encourage surgeons to collaborate closely with radiologists in order to differentiate patients in need for emergency surgery.

## Supplementary Information

Below is the link to the electronic supplementary material.

Supplementary video (MP4 26832 kb)
